# A deep neural network-based approach for seizure activity recognition of epilepsy sufferers

**DOI:** 10.3389/fmed.2024.1405848

**Published:** 2024-07-24

**Authors:** Danial Khurshid, Fazli Wahid, Sikandar Ali, Abdu H. Gumaei, Samah M. Alzanin, Mogeeb A. A. Mosleh

**Affiliations:** ^1^Department of Information Technology, The University of Haripur, Haripur, Khyber Pakhtunkhwa, Pakistan; ^2^College of Science and Engineering, School of Computing, University of Derby, Derby, United Kingdom; ^3^School of Computing Sciences, University of East Anglia, Norwich, United Kingdom; ^4^Department of Computer Science, College of Computer Engineering and Sciences, Prince Sattam bin Abdulaziz University, Al-Kharj, Saudi Arabia; ^5^Faculty of Engineering and Information Technology, Taiz University, Taiz, Yemen; ^6^International University of Technology Twintech, Sana’a, Yemen

**Keywords:** deep learning, deep neural network, electroencephalogram, epilepsy disability, epilepsy detection, seizure activity recognition

## Abstract

Epilepsy is one of the most frequent neurological illnesses caused by epileptic seizures and the second most prevalent neurological ailment after stroke, affecting millions of people worldwide. People with epileptic disease are considered a category of people with disabilities. It significantly impairs a person’s capacity to perform daily tasks, especially those requiring focusing or remembering. Electroencephalogram (EEG) signals are commonly used to diagnose people with epilepsy. However, it is tedious, time-consuming, and subjected to human errors. Several machine learning techniques have been applied to recognize epilepsy previously, but they have some limitations. This study proposes a deep neural network (DNN) machine learning model to determine the existing limitations of previous studies by improving the recognition efficiency of epileptic disease. A public dataset is used in this study and classified into training and testing sets. Experiments were performed to evaluate the DNN model with different dataset classification ratios (80:20), (70:30), (60:40), and (50:50) for training and testing, respectively. Results were evaluated by using different performance metrics including validations, and comparison processes that allow the assessment of the model’s effectiveness. The experimental results showed that the overall efficiency of the proposed model is the highest compared with previous works, with an accuracy rate of 97%. Thus, this study is more accurate and efficient than the existing seizure detection approaches. DNN model has great potential for recognizing epileptic patient activity using a numerical EEG dataset offering a data-driven approach to improve the accuracy and reliability of seizure detection systems for the betterment of patient care and management of epilepsy.

## 1 Introduction

Epilepsy is a prevalent neurological condition that affects millions of people worldwide. It is considered a kind of disability, where epileptic patients are considered a category of people with disabilities. Different techniques are used to detect c activities and their shortcomings. EEG is the manual way of diagnosing seizures by pinning many electrodes everywhere on the head, making it difficult to pinpoint where the electrical activity in the brain originates. Additionally, medical professionals’ reading of EEG signals is slow, time-consuming, and subject to human mistakes during the diagnosis process. Machine learning techniques are also used to identify epileptic seizures. Different methods have been adapted for epilepsy detection, such as CNN, K-NN, Naïve Bayes, and DWT, and briefly discussed in the literature of the study. However, most existing state-of-the-art methods are considered complex, time-consuming, and suffer from some limitations in terms of accuracy performance.

**Figure 1:**
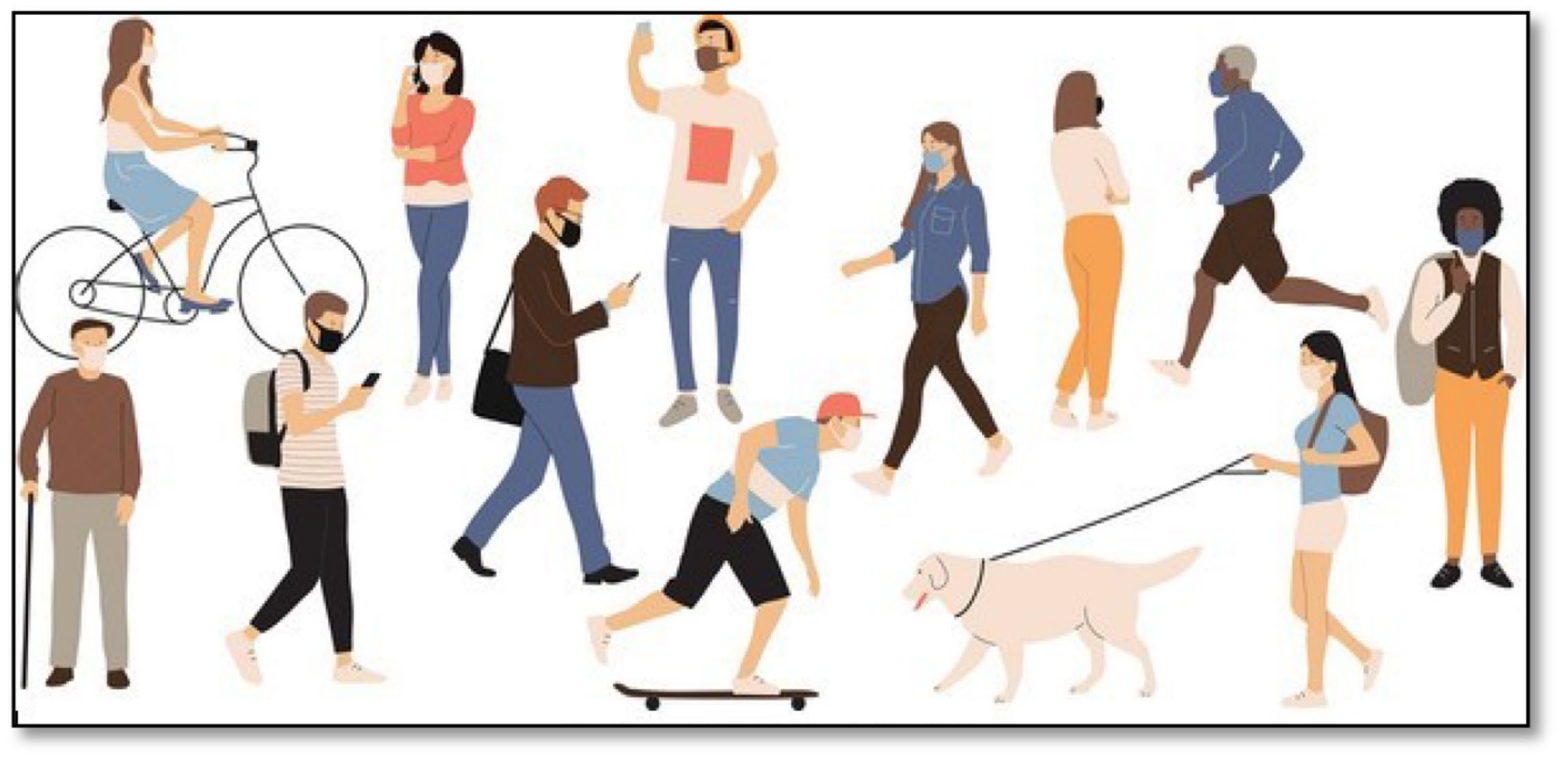
Human activities of daily life (5).

Early epilepsy detection can help society, health sectors, and medical specialists. Human activity recognition (HAR) is the automatic detection of numerous physical actions people perform daily. It is used to identify the actions that are carried out by a person, given a set of observations of themselves and the nearby environment. Activity recognition can be attained by exploiting the information retrieved from various sources, such as environmental (1) or body-worn sensors (2). Multiple approaches have adapted dedicated motion sensors in different body parts such as the wrist, waist, and chest. These sensors are primarily uncomfortable for users and do not provide long-term results for activity monitoring, e.g., sensor repositioning after dressing (3). A HAR system aids in the recognition of a person’s activities and the provision of intervention responses. Most activities that keep track of everyday fitness exercises, such as walking, jogging, walking upstairs, and walking downstairs, are done daily. Taking phone calls, sweeping, making food, combing hair, washing hands, brushing teeth, wearing coats and shoes, and writing and reading are all tasks that everyone does daily. Also growing demand for wearable devices with sensing abilities (smart watches, intelligent bands) used to take out important information (4). Figure 1 shows some of the daily activities of human life.

Through wearable devices, human activity recognition (HAR) is currently considered an essential tool for health care in the future. Tracking patient activities not only helps medical professionals to provide hospital care services to patients across any distance with the latest technology of communication and information but also provides facilities for patients to be monitored online (6). The advantages are the prevention of hospitalization, the cost, and improving human health. Patient activity recognition PAR includes monitoring Vital Data (VD) such as blood pressure, pulse, and blood glucose (7).

**Figure 2:**
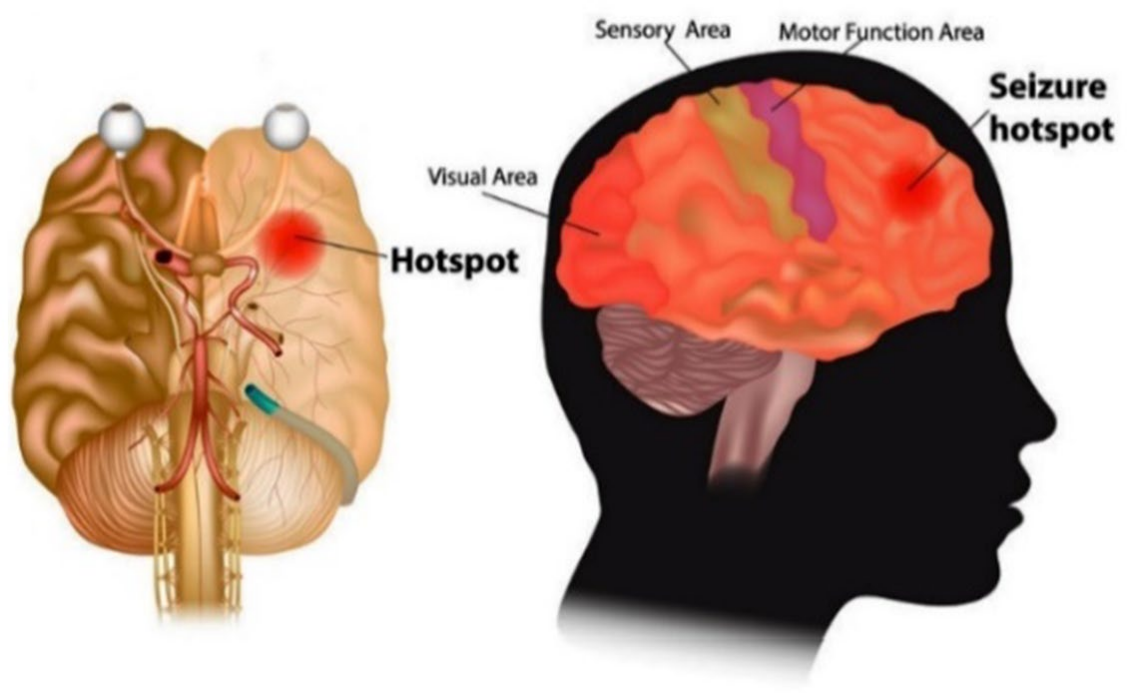
Seizure hotspots in the human brain (10).

Different sensors are used to monitor various activities to improve patients’ health. The developments in wearable and cell phone devices have made it possible to gather information from built-in smartphones and health trackers, including microphones, magnetometers, gyroscopes, GPS, and accelerometers. An epileptic seizure is a usual neurological disorder that happens because of unexpected discharge of neurons of the brain and stress influence. It is a condition distinguished by repeated (two or more) epileptic seizures. A single event is considered as numerous seizures occurring within a 24-h time or an episode of status epilepticus (SE). It is one of the world’s oldest conditions of humankind, and still, it is the most typical neurological condition that affects people of all ages. About 50 million people worldwide have a diagnosis of epilepsy (8). A clinical device, an electroencephalogram (EEG) signal, plays a vital role in diagnosing epilepsy. It gives a photograph of the human brain while doing a cognitive task or even resting. The EEG is gathered by putting electrodes on the patient’s scalp. Then, electro-activity is recorded, produced by the brain, and can identify epilepsy, but this method for examining an EEG signal for epileptic seizure recognition is time-consuming (9). Figure 2 visualizes the hotspot of seizure in the human brain.

Machine learning techniques have been proposed to switch this typical method. There are two fundamental stages of extraction and classification of data involved in machine learning. The traditional system of consulting doctors is time-consuming and more costly, also leading to fatigue-based diagnostic mistakes and subject to the absence of diagnostic facilities in regions of the world where physicians are not available. Recently, machine learning methods have been capable of attaining skilled-level performance in health care and the medical field (11). Different deep learning approaches are used to detect seizures, like support vector machines, convolutional neural networks, and deep convolutional neural networks. Still, these techniques use complex algorithms and image data extracted from EEG.

There are several reasons behind the development of a Deep Neural Network-based method for identifying seizure activity in epilepsy patients. First and foremost, it tackles the pressing issue of prompt and accurate seizure detection, which is necessary for both patient care and efficient treatment. Furthermore, improvements in machine learning—especially in deep learning—present the possibility of very precise pattern identification in EEG data, which might improve detection rates. This strategy also seeks to enhance the quality of life for individuals with epilepsy by facilitating more targeted intervention techniques, which in turn lowers the frequency and intensity of seizures.

This study used a deep neural network (DNN) based model to recognize seizures. Since patterns of EEG seizures differ significantly between patients, it is challenging to recognize seizures. Thus, most of the automated methods that will be discussed in the literature review use complex algorithms and substantial image data sets, which is time-consuming and inefficient. The focus is on creating a model to swiftly and accurately detect epilepsy. Our main aim is to develop a fast and precise system. Through thorough testing and training, we aim to achieve high accuracy while also considering speed. Ultimately, our goal is to improve epilepsy diagnosis, potentially benefiting patients with better and faster care.

## 2 Literature review

Many deep learning and machine learning methods and algorithms are used for the detection of human activities, patient activities, and epileptic patient activities. In this section, some previous work that has been done recently will be discussed. Hassan et al. (12) proposed research on a smartphone inertial sensors-based approach for HAR. Effectual attributes are first taken out via raw data. The attributes contain median, mean, autoregressive coefficients, etc. The attributes are processed through a linear discriminant examination and kernel principal component analysis (KPCA) and (LDA) to make them extra robust. Lastly, the attributes are trained by a Deep Belief Network (DBN) for effective activity detection. The system comprises three central portions: sensing, attribute recognition, and extraction. The sensing part collects the sensor’s information as input to the HAR system. Attribute extraction removes noise to isolate signals. Finally, where DBN is used, a key aspect is modeling actions from attributes via deep learning with an overall accuracy of 95.85%.

Gul et al. (13) researched abnormal human activity recognition as a Tool for Patient Monitoring. The You Look Only Once (YOLO) network, which is based on CNN architecture, is used as a backbone CNN model. To train the CNN model, a large dataset of patient films is constructed by labeling each frame with the positions and behaviors of the patient. For 32 epochs, a CNN model with 23,040 tagged photos of the patient’s actions was used. The model assigned a unique action label and a confidence score for video orders by identifying the recurring action label in each frame. The study found that aberrant action recognition is 96.8% accurate. For patient nursing, the proposed framework can benefit hospitals and elder care homes. Murad et al. (14) performed a study on deep recurrent neural networks (DRNN) and built a model that can capture distant dependencies in variable-length input arrangements. The model has bidirectional, unidirectional, and cascaded structural design, which is built on long short-term memory (LSTM). The approach exceeds other modern methods because it is capable of taking out more particular attributes via deep layers in end-to-end and task-dependent fashion and has an overall accuracy of 96.7%. Uddin et al. (15) performed research on Activity Recognition for Cognitive Assistance Using Body based sensor data and Deep Convolutional Neural Networks in which signals are examined from body wearable sensors for Medicare like gyroscope, ECG, accelerometer, and magnetometer sensors. The deep CNN is trained once attributes are extracted from sensor data using Gaussian kernel-based PCA and Z-score normalization. Lastly, trained deep CNN is utilized to detect activities in examining data. The method provides cognitive aid in wearable sensor-based intelligent medical care systems. The proposed method has an average accuracy of 93.90%.

Ouichka et al. (16) conducted research on prediction of seizures using DNN methods. In which five models (1-CNN, 2-CNN, 3-CNN, 4-CNN, and Transfer learning with ResNet50) for the prediction of epileptic seizures were proposed. The findings show that both methods, one using a fusion of three CNNs (3-CNN) and the other using four CNNs (4-CNN), achieve an accuracy of 95%. Specifically, the 3-CNN method yields an accuracy of 95.0%, a recall of 94.5%, and an F1-score of 95.0%. The 4-CNN method provides an accuracy of 95.5%, a recall of 95.5%, and an F1-score of 95.0%. Ibrahim et al. (17) presented two patient-specific CNN models for prediction and detection of seizure in which spectrogram images of EEG signal segments was used. The third CNN model is designed for patient non-specific scenarios and can classify two and three EEG signal states. It operates effectively on both spectrogram and PSR images of EEG segments. Experiments showed the highest classification performance when using PSR images, due to their superior representation of EEG signals. In contrast, the first two models are suitable for patient-specific uses, but their reliance on spectrogram images somewhat restricts their performance.

Poorani et al. (18) performed a research on a one-dimensional, patient-specific scheme for detecting epilepsy seizures addresses binary classification (seizure vs. non-seizure). The 1D-CNN and CNN-LSTM models offer a computationally efficient approach by processing EEG data through pooling and dense layers. Abderrahim et al. (19) conducted an experiment in which they introduces four models: S-CNN, Modif-CNN, CNN-SVM, and Comb-2CNN, each demonstrating high accuracy in predicting epileptic seizures. The Modif-CNN model stands out with an impressive accuracy rate of 97.96%, making the results from all models both promising and interesting.

The presented study also addresses the challenges identified and some limitations of recent studies and machine learning techniques such as many models struggle to handle EEG data in real time and need large amounts of computing power. Additional problems include handling undesired data in the EEG, individual variations in seizure patterns, and an imbalance in data classes. Specifically for other deep learning models Long-term dependency maintenance is a hurdle for RNNs, non-image dataset adaptation may be a barrier for CNNs, training and parameter optimization are issues for RL so the current model that is using to identify Epileptic activities by using multiple hidden layers that allows to learn complex patterns and data representation the depth of these layers allows to capture the complicated features resulting in enhanced performance. DNN algorithm is more efficient because of its computational complexity, deep architecture and its ability to learn complicated patterns from the data as Compare to other deep learning models.

## 3 Problem identification and solution

There are various methods used to detect epileptic seizures; one of the most common and manual ways is EEG, which is a very time-consuming process. Computer-aided diagnosis methods, automatic detection, deep learning, and machine learning methods exist. The conventional technique of identifying different brain disorders has been inspected manually for centuries. Still, those manual methods have some limitations, such as inaccuracy, slow diagnosing process, and various outcomes of the same inputs. Manual identification needs more resources and time. So, to achieve high accuracy and fast diagnosis, computer-aided disease detection methods have been used for the last few decades. This method will assist medical professionals in the clarification of medical imaging. Medical computer-aided diagnosis methods are limited by noise, fuzziness, and uncertainty in medical images, so such limitations may affect decisions of disease diagnosis while determining the disease type. The main idea of this research is to detect epileptic seizures using a Deep Neural Network (DNN), which is more powerful and optimistic. A simple numerical model that is built on deep learning has applications in the fields of bioinformatics, healthcare, and computer science. The personal monitoring system for the detection of epilepsy with high accuracy is becoming popular for the improvement of human life. Researchers can achieve their targeted objectives and improve their expertise through this research. In the current study, the DNN model contains several layers of neurons that build up an output layer.

## 4 Proposed methodology

**Figure 3:**
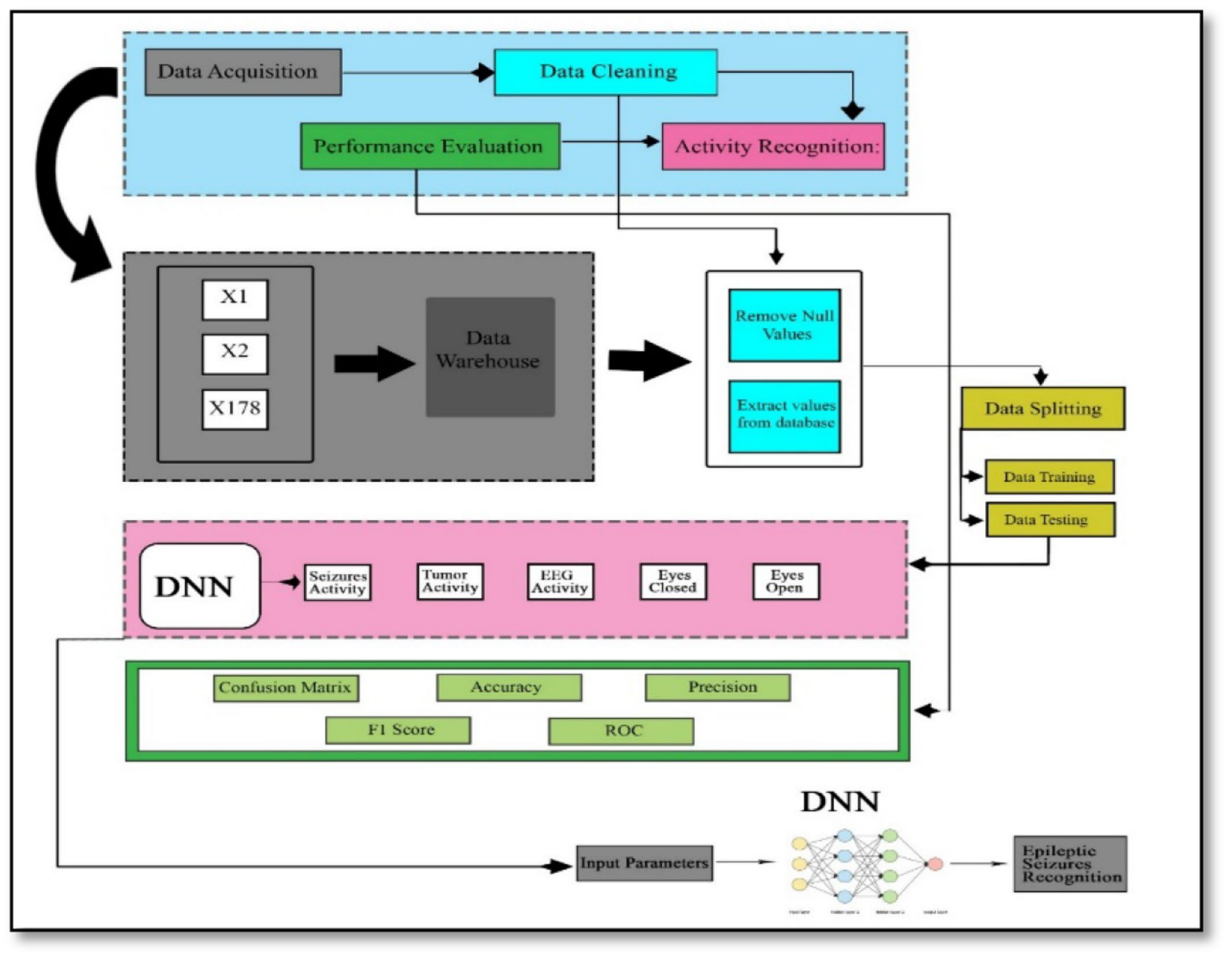
Adopted methodology.

The proposed method consists of four main stages, illustrated in Figure 3. Initially, data acquisition involves collecting the necessary data. This is followed by the data cleaning stage, where irrelevant or redundant features are eliminated to ensure the dataset is optimized for further analysis. Once cleaned, the dataset is divided into two subsets: one for training the model and the other for testing its performance.

In the activity recognition phase, a deep neural network is employed to identify brain activities related to seizures. This involves the model learning patterns and distinguishing between different types of brain activity. Finally, in the performance evaluation phase, the model’s effectiveness is assessed using various metrics. These metrics include the F1-score, which balances precision and recall, precision itself, the confusion matrix that shows the performance of the classification, accuracy indicating the proportion of correctly classified instances, and the Receiver Operating Characteristic (ROC) curve, which illustrates the true positive rate against the false positive rate across different threshold values. This structured approach ensures that each phase contributes to building a robust and reliable model for recognizing epileptic seizures, with thorough evaluation to validate its performance.

### 4.1 Data loading

The data of this study is publicly available and uploaded to the model for cleaning, splitting and classification. After uploading the historical data, the valuable data will be extracted, and then irregular, null, garbage, and inconsistent values will be eliminated, which may lead to many difficulties. Data cleaning removes unwanted features that do not belong to the proposed study. In the next stage, data transformation is done, in which the raw data is turned into a format or structure that is more suited for the model or algorithm.

### 4.2 Data splitting

The data is split into two parts: the first part of the dataset is used for training, and the remaining part will be used for data testing. The proposed model will split DNN’s dataset into different training and testing ratios to achieve high accuracy.

#### 4.2.1 Training set

The data samples are used to fit the model, and a subset of the dataset is used to train the model (in the context of neural networks, calculating weights and biases). The model sees and learns this data, allowing it to improve its parameters.

#### 4.2.2 Test set

The data set objectively evaluates a final model’s fit to the training data. It is used once the model has been adequately trained with training and validation.

### 4.3 Model architecture

ANN’s model architecture includes the creation of layers, which are input layers, dense layers, and output layers. Each neuron in the dense layer receives input from all neurons in the previous layer, making it a deep-connected neural network layer. The thick layer is revealed to be the layer that is most usually utilized. The size of the input layers and output layers are also defined in this section.

### 4.4 Model compilation

Compilation is the last stage in the model creation process. The model will be ready to move to the training and testing phase at this stage. The model compilation uses some functions, such as the loss function, to find errors or deviations in the learning procedure. Moreover, the optimizer is used to optimize the weights of the inputs by comparing the loss function and prediction. The evaluation metrics are applied to evaluate the model’s performance.

### 4.5 Model training

The training set consisted of sample output data and the input data sets that affect the outcome. The training model is utilized to process the input data using the algorithm to match the processed result to the sample output. NumPy arrays using the fit function are used to train models. The main aim of the fit function is to evaluate the model during the training stage (20).

### 4.6 Model testing

After the training model moves toward the testing phase, testing of the model is the process of analyzing a fully trained model’s performance on a testing set. The testing set is a collection of samples separated from the training and validation sets, but it has the same probability distribution as the training set (21).

### 4.7 Model evaluation

In this stage, performance evaluation will be done to improve the system. Confusion matrix, F1-score, Precision, recall, and accuracy in a rigorously statistical manner are the parameters utilized for performance evaluation.

#### 4.7.1 Confusion matrix

A Confusion Matrix is an n x n matrix used to assess the model’s classification performance, where N represents the number of target classes. The matrix differentiates the actual values from the machine learning model’s predictions. This gives us a clear picture of how efficiently our classification method works and the types of errors it generates (22).

#### 4.7.2 Accuracy

Model accuracy is a metric for determining which model is the most effective in detecting patterns and correlations among variables in a dataset using training or input data. The greater a model’s generalization to ‘unseen’ data is, the more accurate insights and predictions it can deliver, and hence the additional commercial value it can provide. The accuracy of classification models is one of the factors to consider while evaluating the (23). Accuracy represents the percentage of correct predictions made by our model. Equation (1) below is the formal definition of:


(1) $$\mathrm{Accuracy}=\frac{\mathrm{Number of right predictions}}{\mathrm{Total number of predictions}}\;$$

Equation (2) below determined binary classification accuracy regarding negatives and positives.


(2) Accuracy=TP+TN/(TP+TN+FP+FN)

TP stands for True Positives, TN stands for True Negatives, FP stands for False Positives, and FN stands for False Negatives.

#### 4.7.3 Precision

Precision is a statistic that measures the accuracy of a machine learning model’s positive prediction. Precision (i.e., the total number of true positives plus the number of false positives) is the ratio of true positives to total positive predictions as shown in Equation (3) below (24).


(3) $$\mathrm{Precision}=\frac{\mathrm{True Positive}\;\left(\mathrm{TP}\right)}{\mathrm{True Positive}\;\left(\mathrm{TP}\right)+\mathrm{False Positive}\left(\mathrm{FP}\right)}\;$$

#### 4.7.4 Recall

The model’s recall indicates how successfully it finds True Positives. As an outcome, recall tells us how many patients we correctly identified as having illness out of the total number of patients with disease (25). Mathematically shown in Equation (4) below.


(4) $$\mathrm{Recall}=\frac{\mathrm{True Positive}\;\left(\mathrm{TP}\right)}{\mathrm{True Positive}\;\left(\mathrm{TP}\right)+\mathrm{False Negative}\left(\mathrm{FN}\right)}$$

#### 4.7.5 F1-score

The F1 score represents a balance of precision and recall. The harmonic mean of accuracy and recall is used to compute the F1 score. The harmonic mean is a measure that can be used instead of the arithmetic mean. Calculating an average rate is especially beneficial (26). The average accuracy and recall are computed using the F1-score. Because they are both rates, the harmonic mean makes sense. It is calculated using the Equation (5) below:


(5) $$F1-\mathrm{score}=\frac{2*\left(\mathrm{Precision}*\mathrm{Recall}\right)}{\left(\mathrm{Precision}+\mathrm{Recall}\right)}$$

## 5 Experimental setup

The experiments that are done are related to epilepsy detection using deep neural networks and will be deeply discussed in this section.

### 5.1 System specification

The system that is used in this research is an HP Intel core i5-fourth generation Desktop with 8 GB RAM, 1.90GHz processor, and 500 GB hard drive—Windows 10 64-bit operating system. In the proposed research, Python language is used to simulate Epileptic patient activity recognition. Google COLAB is used to execute the Python code.

### 5.2 Dataset description

The dataset used in this study is publicly available on the KAGGLE platform at the following link: https://www.kaggle.com/datasets/harunshimanto/epileptic-seizure-recognition. The reference’s original dataset is separated into five categories, each containing 100 files, each representing a particular subject/person. For 23.6 s, each file records brain activity. A 4097 of data points are taken from the linked time series. Each data point represents the value of the EEG recording at a certain instant in time. So it has an overall of 500 people, each with 4,097 data points collected over 23.5 s.

All 4,097 data points are split and scrambled into 23 portions, each holding 178 data points for 1 s, with each data point reflecting the amplitude of the EEG recording at a certain point. So, it has 23 × 500 = 11,500 pieces of data (row), each data point containing 178 data points for 1 s (column), and the last column represents the labels y , which are 1, 2, 3, 4, and 5. In column 179, the response variable is y , and the explanatory variables are $X_{1},X_{2}\ldots X_{178}$ . The 178-dimensional input vector’s category is stored in y. In particular, 1, 2, 3, 4, and 5. Seizure activity is recorded. They took an EEG recording from the tumor’s location. They located the tumor in the brain and captured EEG activity in a normal brain region.

Eyes closed, which suggests the patient’s eyes were closed while the EEG signal was being recorded. Also, eyes open refers to the patient’s eyes being open while the EEG signal of the brain is being recorded.

There are 178 EEG characteristics and five potential classes, as mentioned before. The dataset’s purpose is to detect epilepsy from EEG data correctly. There are five classes in the dataset. The class label 1 is for patients who have an epileptic seizure (seizure activity). The other classes, 2, 3, 4, and 5, are for the patients who did not have epileptic seizures (non-seizure activity). In this study, we classify the patients with seizure activity from those with non-seizure activity. Hence, a binary classification task is conducted among class label 1, encoded as class label 1 for patients with seizure activity, and the other classes 2, 3, 4, and 5, encoded as class label 0 for patients with non-seizure activity. Let us specify the dependent variable (*Y*) and independent variables (*X*) to train the model (*f*).

### 5.3 DNN structure

DNNs are capable of identifying complex patterns within data due to their deep architecture, which includes multiple layers of neurons. Proposed model is highly adaptable and can be applied to various tasks, including natural language processing and numerical data processing. This versatility makes them a strong candidate for diverse research applications. When trained with large datasets, DNNs often achieve higher accuracy compared to other models. Their ability to model complex functions and relationships within data is advantageous for tasks requiring precise and detailed analysis. The model consists of three dense layers in which each input layer to each output layer is fully connected. The activation function, rectified linear unit (ReLU), is used in dense layers for the output layer activation function. Sigmoid is used because the model works on binary classification. The dropout with each dense layer temporarily ignores/deactivates the network’s neurons.

### 5.4 Results with 80% training and 20% test sets

**Figure 4:**
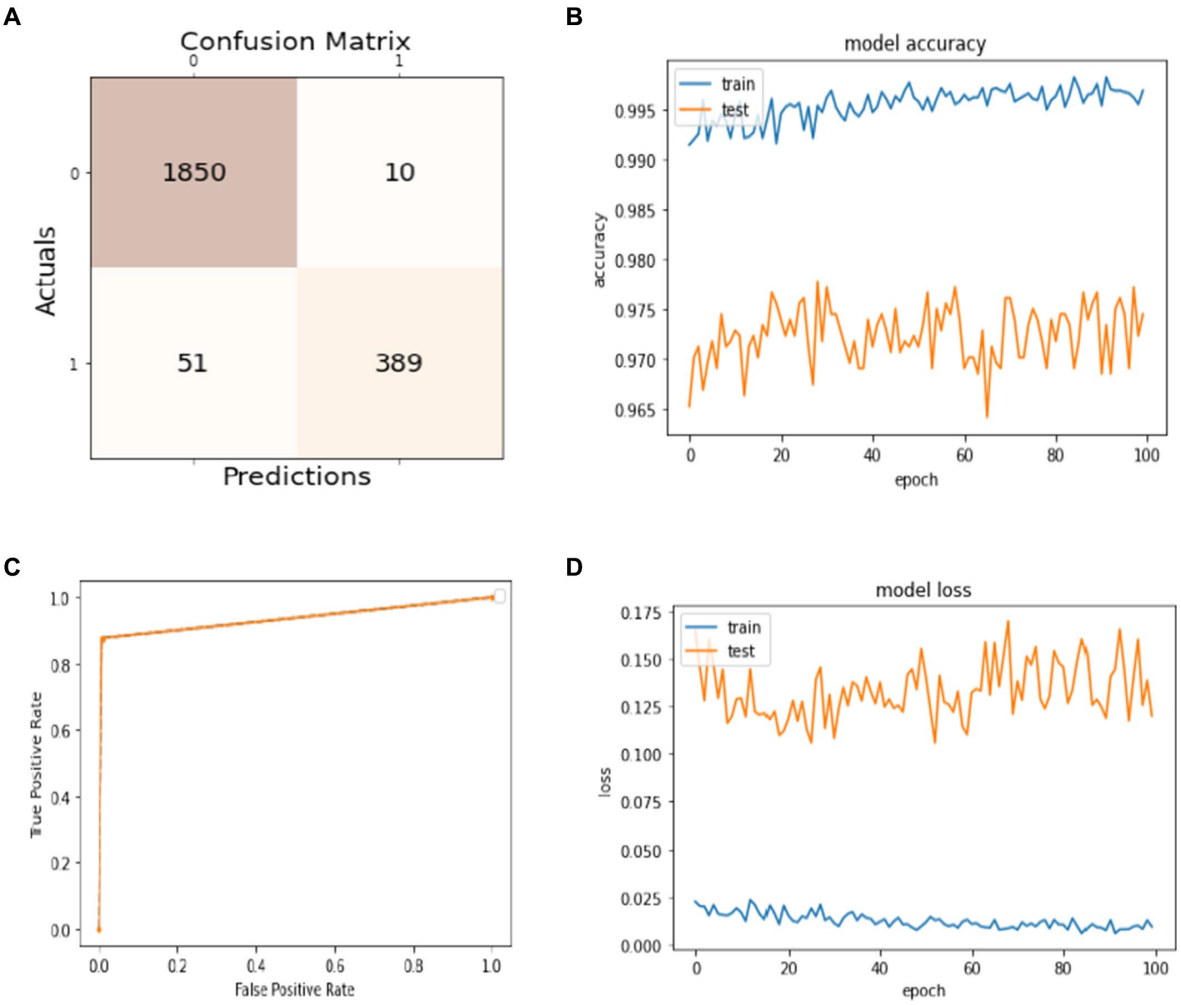
Model training and testing with an 80:20 ratio. **(A)** Confusion matrix; **(B)** model accuracy; **(C)** ROC; **(D)** model loss.

Data splitting is performed with a ratio of 80% for training and 20% for model testing. The results of the experiments are evaluated regarding the true positive examples in the confusion matrix, which are 1850, false positive examples, which are 10. False negative examples, which are 51, and true negative examples, which are 389, as shown in Figure 4A. In Figure 4B, we can see that the accuracy curve of the model for training differs from 99% and above, and the accuracy of testing varies from 96 to 97%. In Figure 4C, the ROC moves from 0.9 of true positive toward 1.0 of false positive rate. In the model loss graph, as shown in Figure 4D, the loss of testing is about 10–17%, and the loss of training is approximately in the range of 0–2%.

The excellence of the developed model can be obtained by the values of precision, recall, F1-score, and accuracy shown in Table 1. The precision of the model is 97% for non-seizure activity, whereas the precision of seizure activity is 98%. The recall results are 99%for non-seizure activity and 88% for seizure activity. Also, we can see that the F1-score for non-seizure activity is 98%, and for seizure activity is 93%, regarding 1860 instances of non-seizure activity and 440 instances of seizure activity.

**Table 1:** Performance evaluation of non-seizure and seizure activity with an 80:20 ratio.

Class label	F1-score (%)	Precision (%)	Recall (%)	Accuracy (%)	Support
0	98	97	99	97	1860
1	93	98	88		440

### 5.5 Results with 70% training and 30% test sets

**Figure 5:**
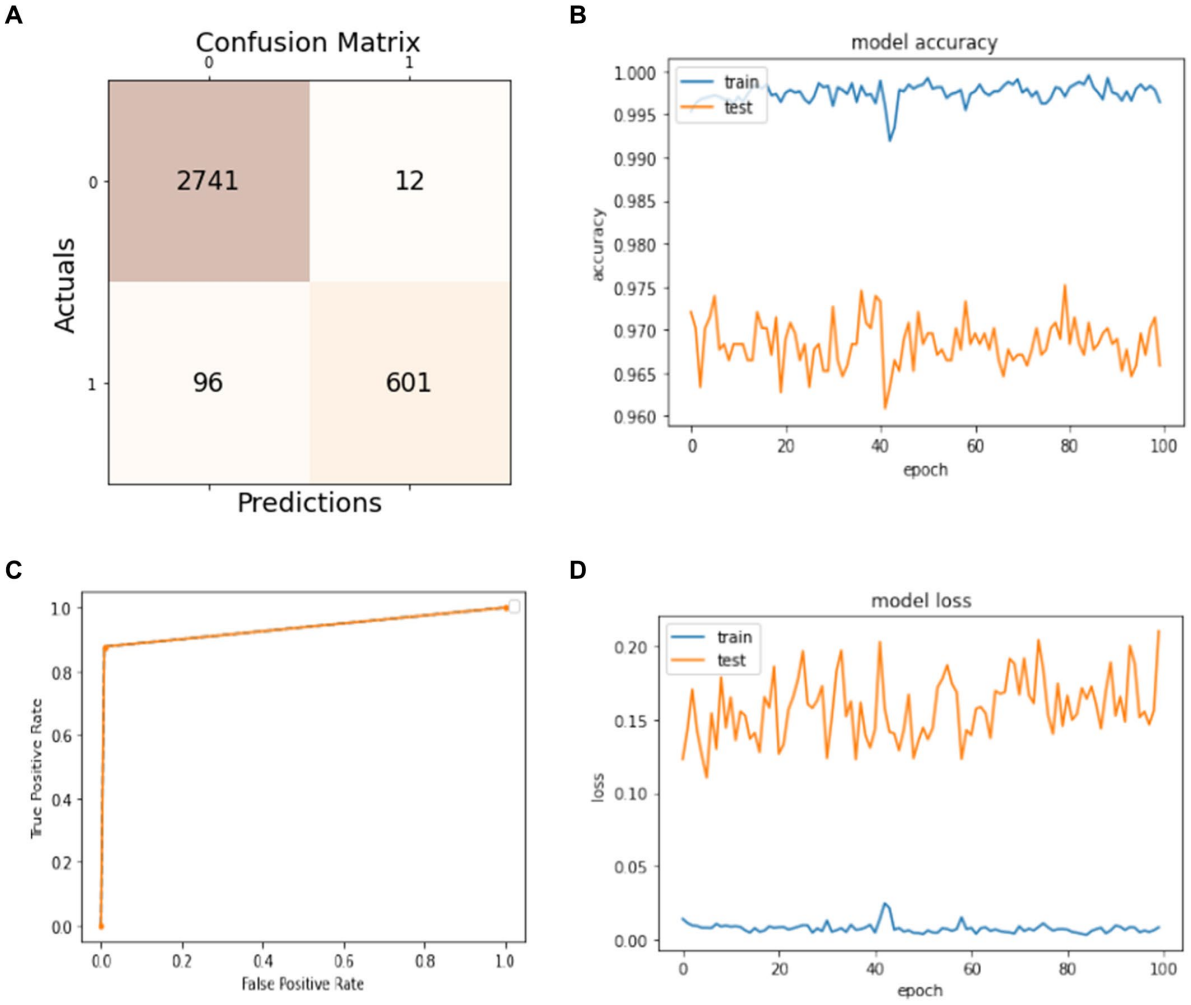
Model training and testing with a 70:30 ratio. **(A)** Confusion matrix; **(B)** model accuracy; **(C)** ROC; **(D)** model loss.

The results of splitting data into 70% for training and 30% for testing the model are discussed in this subsection. As shown in Figure 5A, the confusion matrix displays the first row-wise value to represent the true positive instances, which are patients who do not have epileptic seizures, and the model classifies them correctly as true positive instances. The second value of the confusion matrix is for the false positive instances, which are the model classified incorrectly as patients not having epileptic seizures, but in actuality, they have. The third value of the confusion matrix is several false negative instances, which the model classified as patients having epileptic seizures, but in actuality, they do not have the disease. The last value of the confusion matrix is for the true negative instances that the model classified as patients who have epileptic seizures and have epileptic seizures. In Figure 5B, the blue curve represents the training accuracy of the model, and the orange curve indicates the testing accuracy. It shows that the maximum accuracy of training reaches 99%, and the testing accuracy reaches 97.5% during the different number of epochs. Figure 5C shows the ROC curve that represents the trade-off between specificity (1 – FPR) and sensitivity (or TPR) (27). Basically, it is the relation between the true positive rate and the false positive rate. It shows that when the true positive rate is 0.8, the false positive is 0.0, and when the true positive is 1.0, the true positive is 0.93. Figure 5D visualizes the training and testing model loss, showing how much data is lost at different epochs. The model has 97% overall accuracy, as seen in Figure 5B.

The precision, recall, F1-score, and accuracy values shown in Table 2 show the study’s proficiency. The accuracy of the model is 97% for overall activity recognition. The precision of non-seizure activity is 97 and 98% for seizure activity, whereas the recall for seizure activity is 99% and for non-seizure activity is 86%. The F1-score for non-seizure activity is 98 and 92% for seizure activity. This experiment’s test instances (support) are 2,753 for non-seizure activity and 697 for seizure activity.

**Table 2:** Performance evaluation of non-seizure and seizure activity with a 70–30 ratio.

Class label	F1-score (%)	Precision (%)	Recall (%)	Accuracy (%)	Support
0	98	97	99	97	2753
1	92	98	86		697

### 5.6 Results with 60% training and 40% test sets

**Figure 6:**
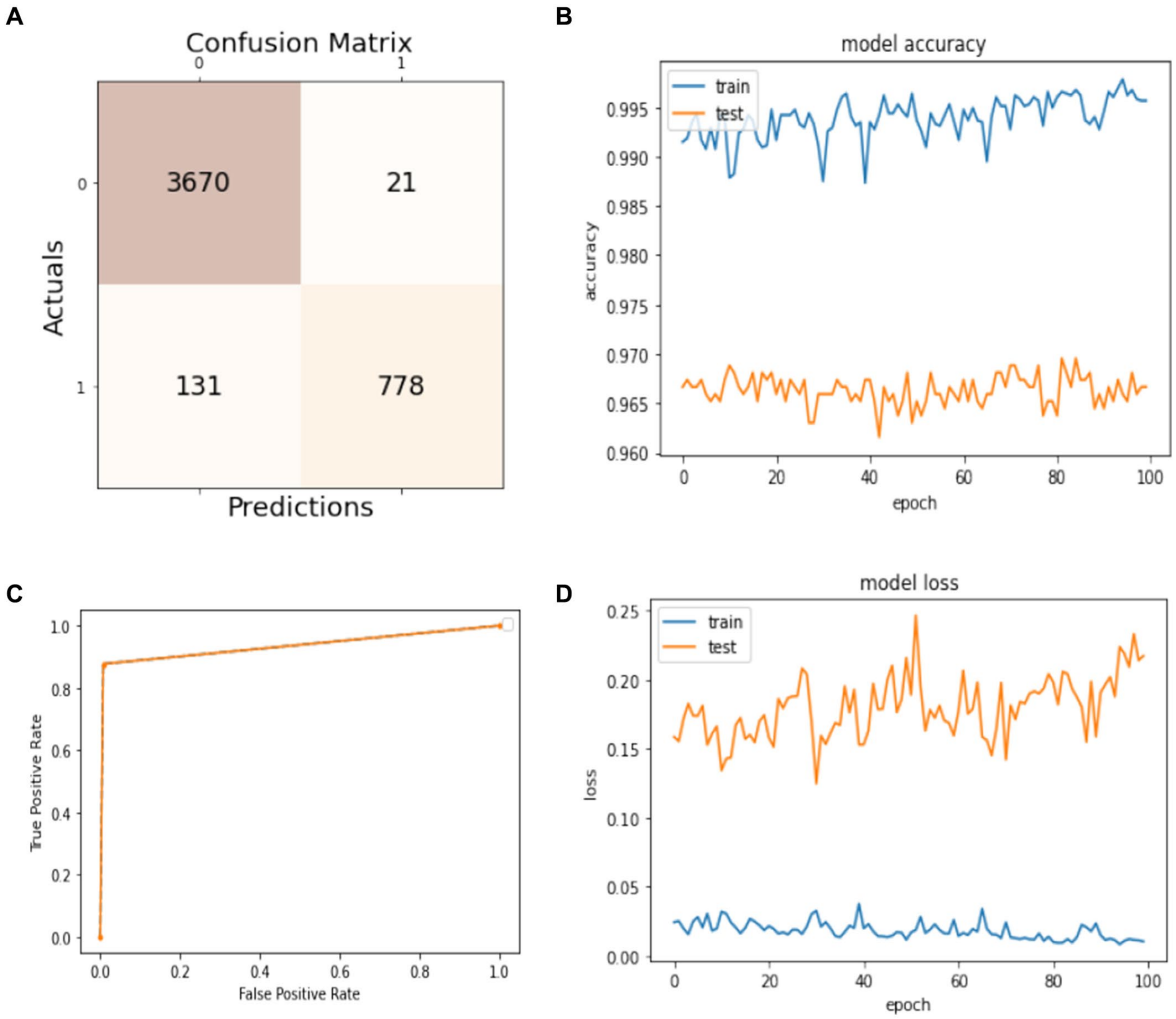
Model training and testing with a 60:40 ratio. **(A)** Confusion matrix; **(B)** model accuracy; **(C)** ROC; **(D)** model loss.

In this subsection, the experiment uses 60% of the dataset for training the model and 40% for the models’ test. The obtained results are presented in Figure 6. The confusion matrix is given in Figure 6A. It shows that 3,670 instances are classified as true positives, 21 instances are classified as false positives, 131 instances are classified as false negatives, and 778 instances are classified as true negatives. Figure 6B visualizes the model accuracy rates during the training process, which are above 99% for training accuracy and between 96 and 97% for testing accuracy. Figure 6C shows the ROC of the model at different numbers of true and false positive rates for the splitting data with a 60:40 ratio. For model loss, Figure 6D shows that the testing loss varies from 15 to 25% and from 0 to 5% for the training loss.

The efficiency of the model can be assessed by the values of precision, recall, F1-score, and accuracy shown in Table 3. The accuracy of the developed model is 96% for classifying both activities, while the precision for non-seizure activity is 97 and 96% for seizure. The recall for non-seizure is 99 and 88% for seizure activity. The F1-score for non-seizure activity is 96 and 91% for seizure activity. The number of instances is 3,663 for non-seizure activity and 937 for seizure activity.

**Table 3:** Performance evaluation of non-seizure and seizure activity with a 60:40 ratio.

Class label	F1-score (%)	Precision (%)	Recall (%)	Accuracy (%)	Support
0	96	97	99	96	3,663
1	91	96	88		937

### 5.7 Results with 50% training and 50% testing sets

**Figure 7:**
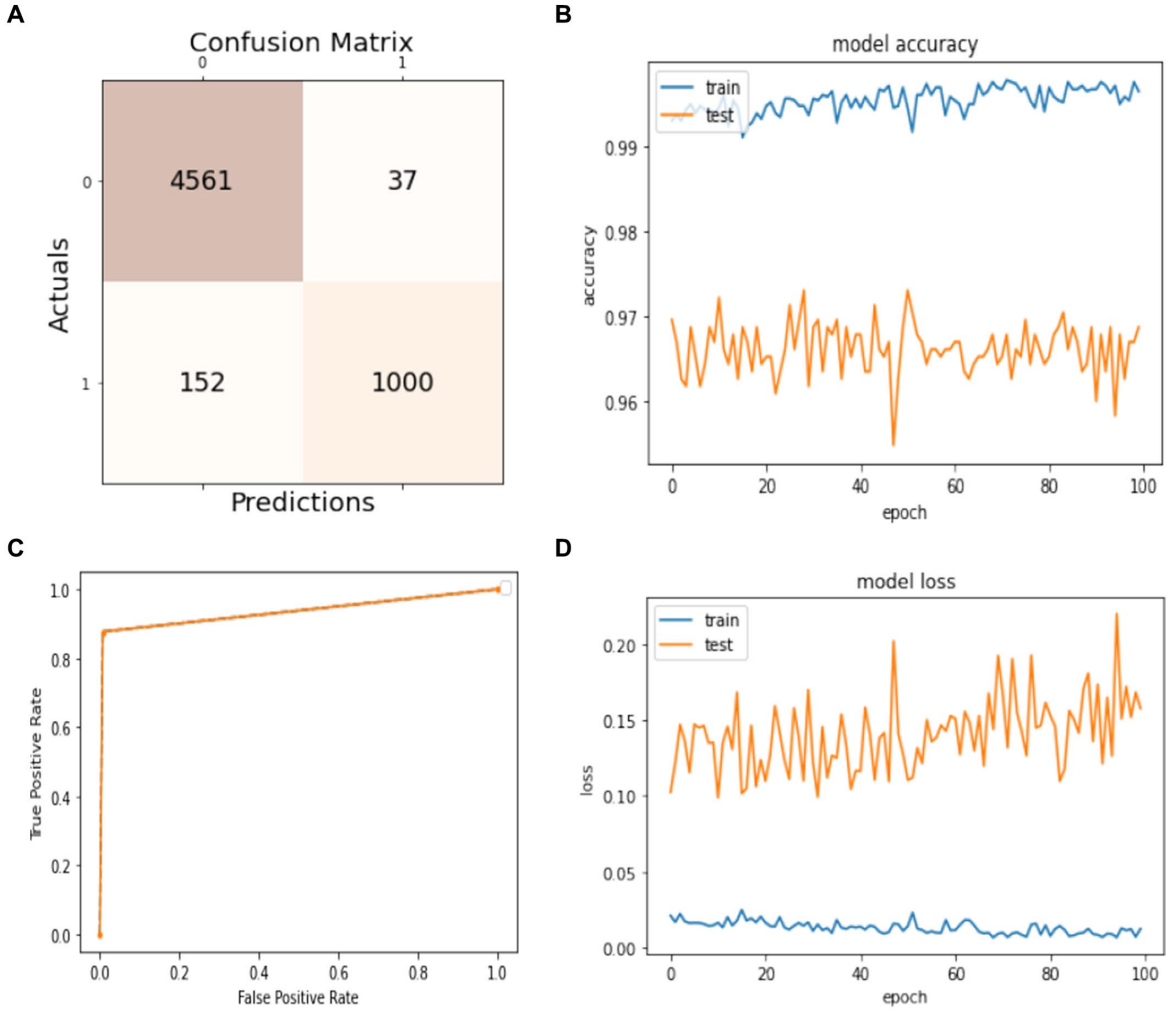
Model training and testing 50–50 ratio. **(A)** Confusion matrix; **(B)** model accuracy; **(C)** ROC; **(D)** model loss.

Figure 7 presents the model’s results trained on 50% of the dataset and tested on the remaining 50%. In Figure 7A, the confusion matrix shows that the number of true positives is 4,561 and the number of false positives is 37, measuring the model’s ability to predict the non-seizure activity truly. The false negative and true negative instances in the confusion matrix, which are 152 and 1,000, mean that the model can predict 152 cases from 1,152 as they have non-seizure activity, but actually, they have seizure activity. Similarly, the model can predict 1,000 instances as they have had seizure activity since 1,152, and they have had seizure activity. The accuracy of training and testing during the training phase are given in Figure 7B. It shows the model’s accuracy fluctuation from 0 to 100 epochs. The same is true for the model’s loss, which is given in Figure 7D. Figure 7C shows the ROC of the model at different numbers of true and false positive rates for the splitting data with a 50:50 ratio.

The results of precision, recall, F1-score, and accuracy are listed in Table 4. It illustrates the effectiveness of the model. We can see that the precision for non-seizure activity is 98% and for seizure is 94%, the recall for non-seizure is 98%, and for seizure is 87%, and the F1-score for non-seizure is 94 and 94% for seizure. The number of test instances (support) is 4,598 for non-seizures and 1,152 for seizure activity. The results of evaluation metrics for the model in overall splitting ratios are presented in Table 5.

**Table 4:** Performance evaluation of non-seizure and seizure activity with a 50–50 ratio.

Class label	F1-score (%)	Precision (%)	Recall (%)	Accuracy (%)	Support
0	94	95	98	95	4,598
1	90	94	87		1,152

**Table 5:** Overall performance of the model with different ratios.

Training	Testing	F1-score (%)	Precision (%)	Recall (%)	Accuracy (%)
80%	20%	93	98	88	97
70%	30%	92	98	86	97
60%	40%	91	96	88	96
50%	50%	90	94	87	95

### 5.8 10-fold cross validation

**Figure 8:**
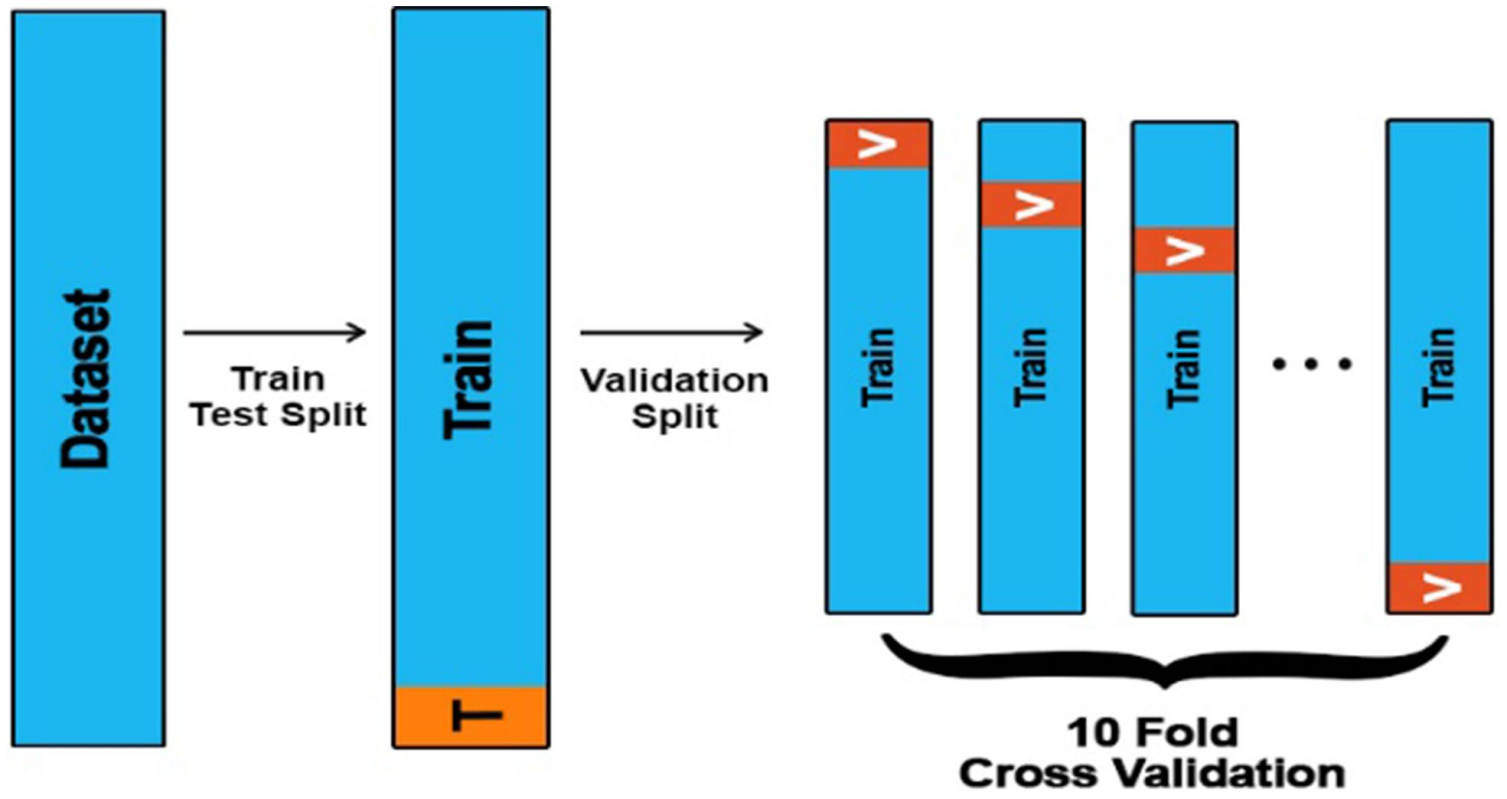
A 10-fold cross-validation technique.

A 10-fold cross-validation technique is applied to the whole dataset to evaluate the model’s performance further, as shown in Figure 8. The total number of instances in the dataset is 11,500. It is divided into 10 equal parts for the 10-fold cross-validation. In each part, 1150 instances are used to test the model. The obtained results are introduced in this subsection. Figure 8 illustrates the strategy of a 10-fold cross-validation technique for splitting the data for training and validation sets.

In Table 6, we present a comparison of different models’ accuracy results using the holdout and 10-fold cross-validation techniques. As we can see, the lowest accuracies are for the logistic regression model, which is 82.5% using a holdout technique, and 80.1% using a 10-fold cross-validation technique, while the highest accuracies are for the proposed model, which is 97% using a holdout technique and 95.5% using a 10-fold cross-validation technique. Also, we can notice that the accuracy of different models using a holdout technique is slightly higher compared to a 10-fold validation technique.

**Table 6:** Accuracy of different models using the holdout and 10-fold cross-validation techniques.

Model	Accuracy of holdout technique (%)	Accuracy of 10-fold validation technique (%)
ANN	95.7	93.4
Naive Bayes	95	94.3
KNN	93.1	91.6
Logistic regression	82.5	80.1
DNN	97	95.5

Table 7 compares different models’ F1-score results using the holdout and 10-fold cross-validation techniques. As we can see, the lowest F1-scores are for the logistic regression model, with 81.5% using a holdout technique and 80.1% using a 10-fold cross-validation technique, while the highest F1-scores are for the proposed model, which is 93% using a holdout technique and 90.5% using a 10-fold cross-validation technique. Also, we can notice that the F1-score of different models using a holdout technique is a little bit higher when compared with a 10-fold validation technique.

**Table 7:** F1-score of different models using the holdout and 10-fold cross-validation techniques.

Model	F1-score of holdout technique (%)	F1-score of 10-fold validation technique (%)
ANN	92.3	90.4
Naive Bayes	89.2	87.3
KNN	90	91.6
Logistic regression	81.5	80.1
DNN	93	90.5

Similarly, in Table 8, we compare the precision results of different models using the holdout and 10-fold cross-validation techniques. As we can see, the lowest precisions are for the logistic regression model, with 81.5% using a holdout technique, and 80.1% using a 10-fold cross-validation technique, while the highest precisions are for the proposed model, with 93% using a holdout technique and 90.5% using a 10-fold cross-validation technique. Also, we can notice that the precision of different models using a holdout technique is slightly higher compared to a 10-fold validation technique.

**Table 8:** The precision of different models using the holdout and 10-fold cross-validation techniques.

Model	Precision of Holdout Technique (%)	Precision of 10-fold Validation Technique (%)
ANN	95	93.4
Naive Bayes	96.4	94.3
KNN	92	91.6
Logistic regression	85.1	83.5
DNN	98	95.5

**Figure 9:**
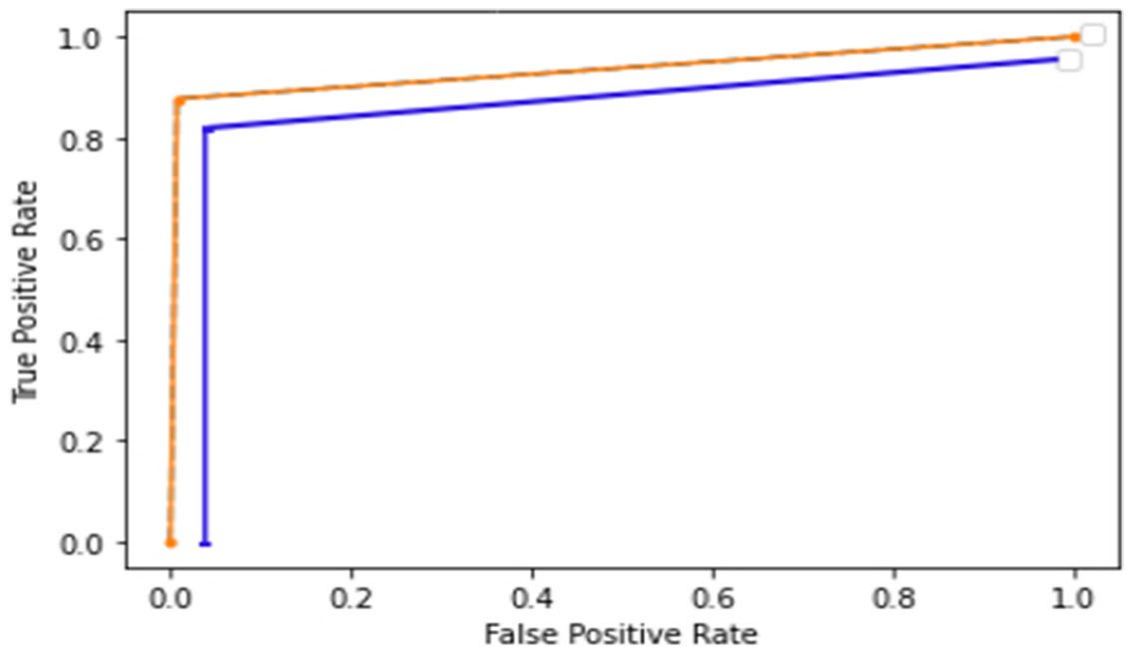
ROC of the proposed model using a holdout and a 10-fold cross-validation technique.

Figure 9 shows the receiver operator characteristic curve (ROC). The orange curve indicates the ROC of the proposed model using a holdout technique. It is shown that when the true positive rate is 0.9, the false positive is 0.0, and when the true positive is 0.93, the false positive is 1.0. The blue curve represents the ROC of the proposed model using a 10-fold cross-validation technique. It is 0.0 when it starts, but when the curve reaches 0.8, the graph achieves a rate of 0.98. The ROC curve presents how well the model can differentiate among positive and negative classes by plotting the true positive rate against the false positive rate at several thresholds. The performance of the model is summarized by a single value by the area under the ROC curve (AUC). When the cost of false positives and false negatives fluctuates, the ROC curve provides a balanced assessment of the model’s performance by taking into account both true positive and false positive rates.

**Figure 10:**
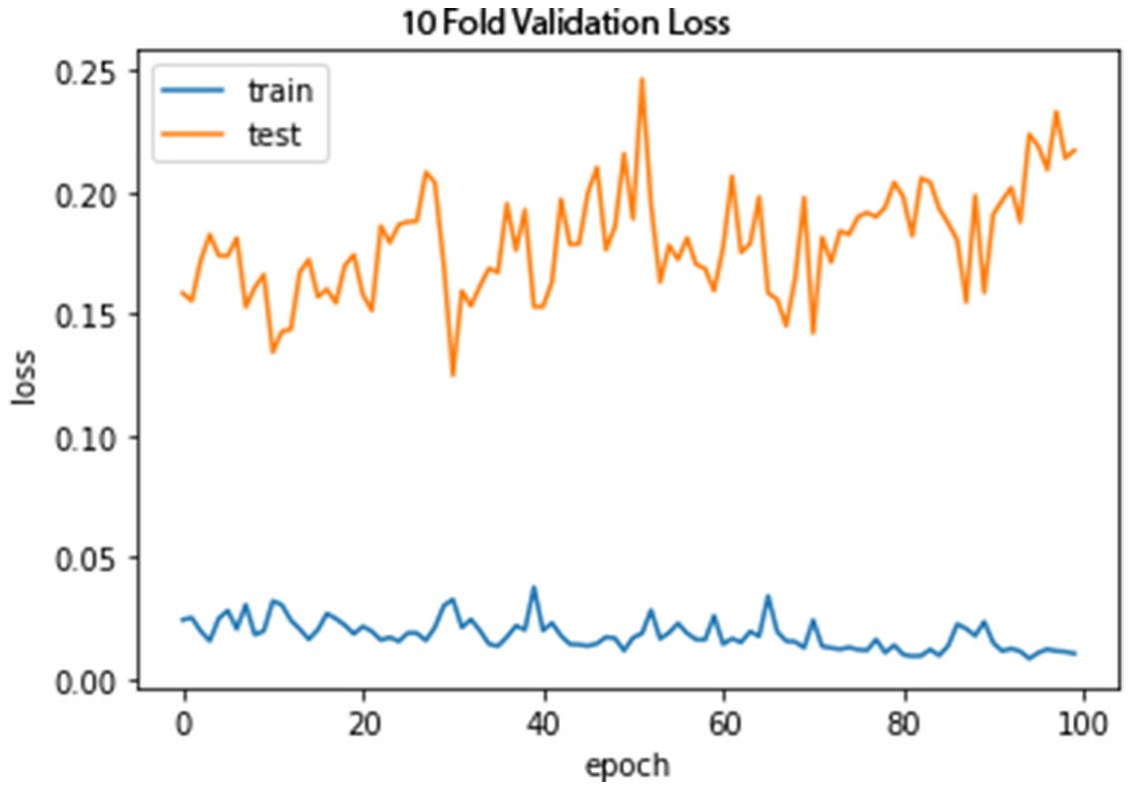
A 10-fold cross-validation loss.

**Figure 11:**
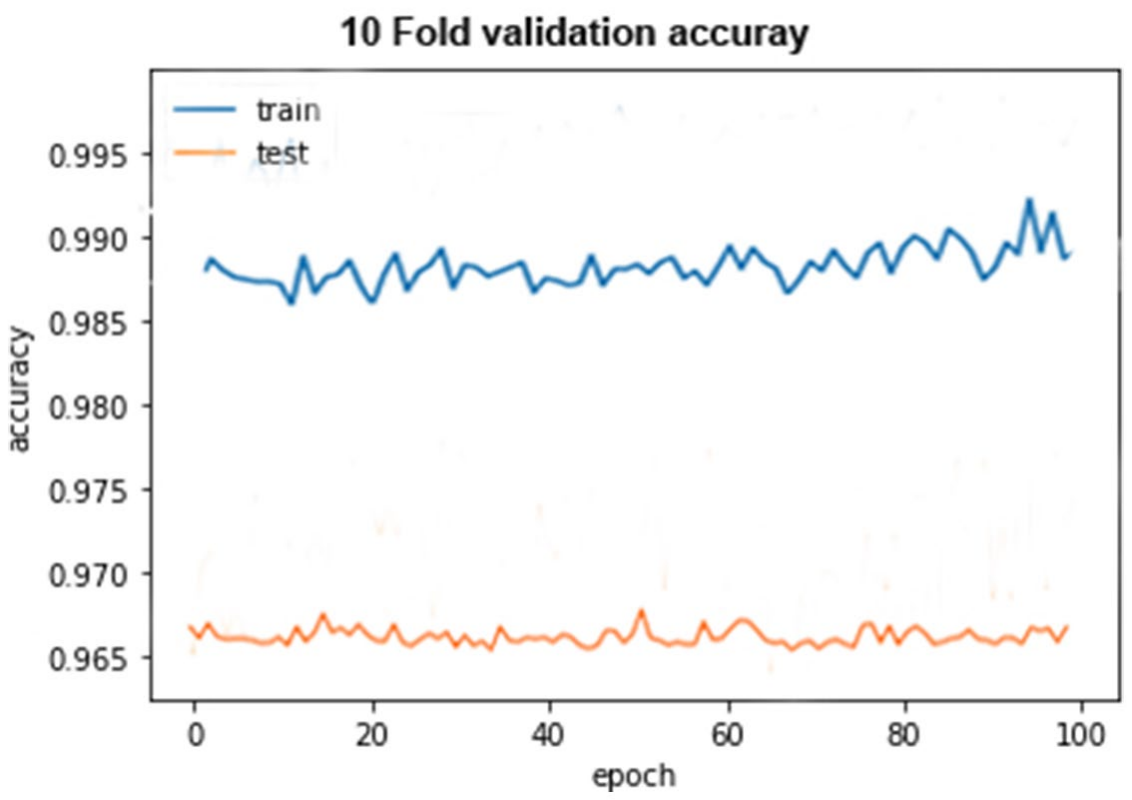
A 10-fold cross-validation accuracy.

Figures 10, 11 show the proposed model’s loss and accuracy using a 10-fold cross-validation technique. The error or model loss graph indicates the overall loss of 10-fold cross-validation during testing and training. In the case of testing, the loss is 0.16% at the first epoch, and it goes higher at 50 and 100 epochs. The loss is 0.23%. For training, the loss is 0.03% on the first epoch and goes higher on the epoch number 40; when it reaches the epoch number 100, the loss is 0.01%.

The accuracy graph for the training of 10-fold cross-validation is shown above as it can be seen that the graph started from 90% accuracy on 0 epoch and remained almost the same at 90 epoch, but an abrupt increase in accuracy can be seen after 90 epoch and achieve 93% accuracy. The accuracy graph for testing of 10-fold cross-validation in which the graph fluctuates between 65 and 100%.

## 6 Discussion

The present study aims to achieve high accuracy by using a numerical data set for our model. The model is trained and tested using different dataset ratios for the best results. Before this study, most of the previous methods used image data sets to execute their research work, but in this study, historical numerical data was employed, which is not complex compared to other methods. Furthermore, a binary classifier (non-seizure or seizure) is used, which does not predefine more specific seizure categories to provide a more generalizable classifier. The DNN algorithm has more than one hidden layer between the input and output layers; the data will be passed through these hidden layers’ functions, in which the function applies weights to the inputs and sends them as the output using an activation function. The activation function used in this study is Sigmoid.

## 7 Comparative analysis

This section will compare the proposed model with the other machine learning approaches concerning accuracy, precision, F1-score, and recall. The comparison of machine learning models with different training and testing ratios, i.e., 80–20%, 70–30%, 60, −40%, and 50–50%, will be done through graphs and tables.

### 7.1 Accuracy

**Figure 12:**
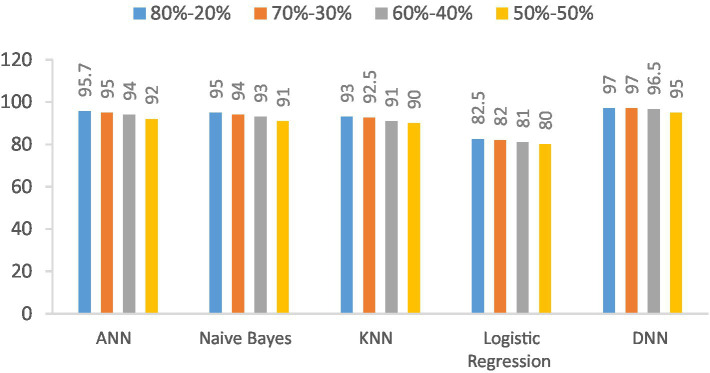
Visualization of accuracy comparison results in the percentage of the proposed DNN model with other models at different splitting ratios.

The accuracy of the proposed DNN model is compared with the other models, such as Logistic regression, KNN, ANN, and Naïve Bayes, using different splitting ratios as given in Table 9 and visualized in Figure 12. We can see that the proposed DNN model achieves the highest accuracy result compared to other models. Despite a general decline in accuracy across all models when the training data is reduced, the DNN model exhibits notable resilience, maintaining comparatively high accuracy even with a balanced 50–50 data split. This suggests that the DNN model is capable of delivering strong performance even with a smaller amount of training data.

**Table 9:** Accuracy (%) comparison results in the percentage of the proposed DNN model with the other models at different splitting ratios.

Model	80–20%	70–30%	60–40%	50–50%
ANN	95.7	95	94	92
Naive Bayes	95	94	93	91.5
KNN	93	92.5	91.5	91
Logistic regression	82.5	82	81	80
DNN	97	97	96.5	95

### 7.2 F1-score

**Figure 13:**
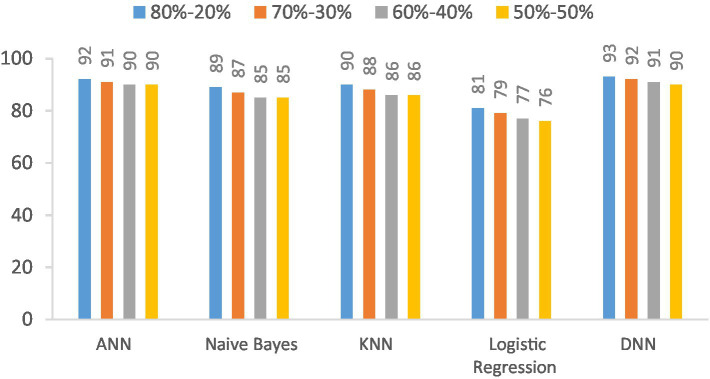
Visualization of F1-score comparison results in percentage of proposed DNN model with other models at different splitting ratios.

The F1-score of the proposed DNN model is compared with the other models, such as Logistic regression, KNN, ANN, and Naïve Bayes, using different splitting ratios as given in Table 10 and visualized in Figure 13. Logistic Regression consistently shows the lowest F1-scores for all data splits, indicating its limited effectiveness for this task. On the other hand, while ANN, KNN, and Naïve Bayes deliver decent results, they still fall short compared to the performance achieved by the DNN model.

**Table 10:** F1-score (%) comparison results in a percentage of the proposed DNN model with other models at different splitting ratios.

Model	80–20%	70–30%	60–40%	50–50%
ANN	92	91	90	90
Naive Bayes	89	87	85	85
KNN	90	88	86	86
Logistic regression	81	79	77	76
DNN	93	92	91	90

### 7.3 Precision

**Figure 14:**
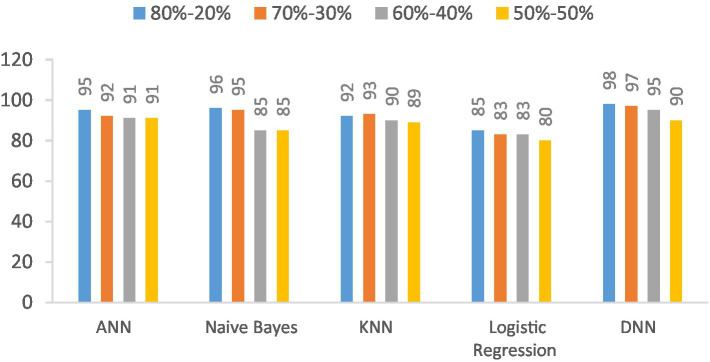
Visualization of precision comparison results in a percentage of the proposed DNN model with other models at different splitting ratios.

We examine the precision of the proposed Deep Neural Network (DNN) model in contrast to several established models: Artificial Neural Network (ANN), Naïve Bayes, K-Nearest Neighbors (KNN), and Logistic Regression. This evaluation encompasses various data splitting ratios, including 80–20%, 70–30%, 60–40%, and 50–50%.as shown in Table 11 and Figure 14. The values will be changed when the training and testing data ratios are changed.

**Table 11:** Precision (%) comparison results in a percentage of the proposed DNN model with other models at different splitting ratios.

Model	80–20%	70–30%	60–40%	50–50%
ANN	95	92	91	91
Naive Bayes	96	95	85	85
KNN	92	93	90	89
Logistic regression	85	83	83	80
DNN	98	97	95	90

### 7.4 Recall

**Figure 15:**
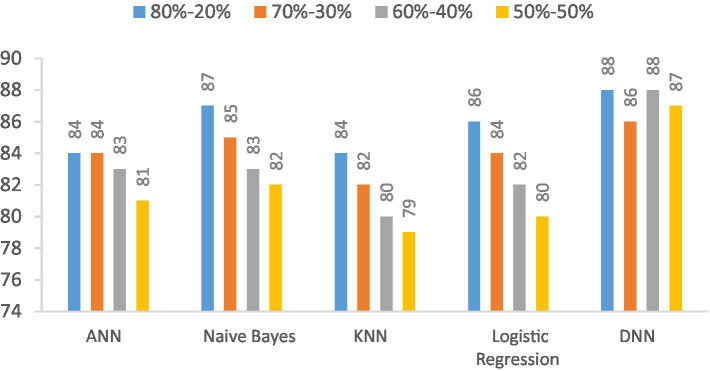
Visualization of recall comparison results in the percentage of the proposed DNN model with other models at different splitting ratios.

In this section, we investigate the recall capabilities of the proposed Deep Neural Network (DNN) model when compared to alternative models across diverse data splitting ratios. The outcomes are illustrated in Table 12 and Figure 15. Recall assesses a model’s proficiency in correctly recognizing all pertinent instances among the total relevant instances. As we manipulate the proportions between training and testing datasets, the figures in the table will adapt accordingly.

**Table 12:** Recall (%) comparison results in the percentage of the proposed DNN model with other models at different splitting ratios.

Model	80–20%	70–30%	60–40%	50–50%
ANN	84	84	83	81
Naive Bayes	87	85	83	82
KNN	84	82	80	79
Logistic regression	86	84	82	80
DNN	88	86	88	87

## 8 Conclusion

The primary objective of this study is to optimize the accuracy and performance of our research outcomes. To accomplish this, we have employed a sophisticated deep neural network (DNN) algorithm while systematically manipulating the ratios of training and testing datasets to discern optimal results. The results showed substantial advancements over previous research endeavors, boasting a remarkable 97% accuracy rate, a precision rate of 98%, an F1-score of 92%, and a recall rate of 80%. Furthermore, our commitment to robust validation methodologies is evident in applying a rigorous 10-fold cross-validation technique designed to further enhance the model’s performance and bolster its reliability across the dataset. Integrating EEG data with other physiological measurements, such as heart rate and movement data, may enhance the accuracy of seizure detection. Future research could investigate methods for combining these diverse data types to utilize the unique benefits of each. Additionally combining the seizure detection system with electronic health records to enhance patient history tracking and care management could also be Upcoming research.

## Data availability statement

Publicly available datasets were analyzed in this study. This data can be found at: https://www.kaggle.com/datasets/harunshimanto/epileptic-seizure-recognition.

## Author contributions

DK: Conceptualization, Data curation, Formal analysis, Investigation, Validation, Visualization, Writing – original draft, Writing – review & editing. FW: Conceptualization, Methodology, Project administration, Resources, Software, Supervision, Writing – original draft, Writing – review & editing. SiA: Conceptualization, Data curation, Investigation, Methodology, Software, Supervision, Validation, Visualization, Writing – original draft, Writing – review & editing. AG: Conceptualization, Data curation, Investigation, Methodology, Project administration, Resources, Software, Supervision, Validation, Visualization, Writing – original draft, Writing – review & editing. SaA: Formal analysis, Investigation, Methodology, Project administration, Resources, Software, Supervision, Visualization, Writing – original draft, Writing – review & editing. MM: Conceptualization, Investigation, Methodology, Project administration, Resources, Supervision, Visualization, Writing – original draft, Writing – review & editing.
